# A Jewish Perspective of the Theory of Evolution – A Response

**DOI:** 10.5041/RMMJ.10045

**Published:** 2011-04-30

**Authors:** Avraham Steinberg

**Affiliations:** Director, Medical Ethics Unit, Senior Pediatric Neurologist, Shaare Zedek Medical Center, Jerusalem, Israel

In my article on a Jewish perspective of the theory of evolution^1^ I wrote:

“There is great zealotry in all debating parties with mutual intolerance of ideas and concepts, disrespect toward opposing opinions and positions, and usage of very harsh language. This prejudiced approach usually does not allow for a reasonable debate”.

Unfortunately, Dr Jacob in a response to my article entitled “Reflections on Darwinian Evolution – Is there a Jewish Perspective?”^2^ has followed that path. Once again we find sentences and statements such as “what he calls “a creationist fundamentalist view masquerading as “whitewashing apologetics”, “pays only lip-service”, etc. These “pearls” point to the problematic approach of some scientists who cannot bear any criticism of the “sacred religion” of Darwinian theory and have great difficulties to explain many fundamental issues in this theory; hence, by mocking opposing views they apparently find some comfort in their beliefs, some of which are – as pointed out in my article – scientifically completely unfounded.

By contrast, I fully respect opposing views even if I disagree with them, such as those which are proposed in Dr Jacob’s article, and hence I shall relate briefly to *some* of Dr Jacob’s arguments in a factual manner.

Dr Jacob argues that “science does not begin with facts; rather, all experimentation begins with the premise ‘Let us assume that ...’. In short, science starts with theories and concepts about the physical world”. Dr Jacob is right that experimentation *may* begin with theories and concepts, but he is obviously wrong by stating that *all* experimentation begins in such a way. In fact, science can – legitimately – start from facts and build up toward theories and vice versa. My point, however, was apparently missed or misunderstood by Dr Jacob: in either way theories remain only theories until proven experimentally. In my article I phrased it in the following way: “The fundamental aspect of modern science is the experimentally proven data under controlled conditions which confirm or reject the theoretical hypotheses about how phenomena work”. The theory of the ancient scientists about the sun turning around the earth as well as the theory about the earth being flat with four corners were accepted theories by all scientists. I assume that if Dr Jacob was living then he would have adopted these theories as “facts” and fought against whoever would have said differently, since those facts were “confirmed then to such a degree that it would have been perverse to withhold assent”. However, these theories were experimentally unproven and with time were proven to be totally wrong. The fact that ancient scientists had a theory about the world and the universe did not prove it to be true because it was not validated by experimental proofs. The example of the apple and gravitation given by Dr Jacob is obviously ridiculous, because the fact that apples fall by gravitation has been proven time and again, and hence gravitation is a scientifically proven theory. Nonetheless, even this theory underwent significant modification by the theory of relativism, pointing again to the fact that scientific theories are constantly changing. Indeed, this universally accepted notion that scientific theories come and go seems in the eyes of scientists like Dr Jacob not to apply to the theory of evolution. This theory, even though not scientifically proven in some of its major components, is considered by many current scientists such as Dr Jacob as immortal. Some of the scientists believe in the absolute truth of this theory, and they do not want to be confused by the lack of experimentally proven major components of the theory. Such an approach, however, is contrary to basic scientific thinking and methodology, and in fact undermines the very foundation of modern science.

Dr Jacob further argues that evolution is a fact as well as it is a theory. He claims that “Humans evolved from apelike ancestors whether they did so by Darwin’s proposed mechanism or by some other yet to be discovered”. He further compares this “fact” to the fact of falling apples, whether it happens by Newton’s or by Einstein’s theories. In my view, this comparison of facts is an excellent example of a distorted scientific approach: I can see for myself every day the *fact* that apples fall from trees, and so can Dr Jacob and millions of human beings today and in the past. This fact can be explained either by Newton’s or by Einstein’s theory. However, neither I, nor Dr Jacob, nor anyone ever in history has ever seen the transition of an apelike creature to a human being. Moreover, scientists have never been able to use evolutionary mechanisms to transform one species into another. Also, the time-line for generating all of the necessary mutations to get to our point in time is several fold longer than what science claims is the age of life. In response, science must invent “periods of accelerated mutations” of which there is little proof. Hence, this was, and stays, a *theory,* not a *fact,* based on very problematic assumptions, which I discussed at length in my article, and which Dr Jacob, for reasons unknown to me, fails to address at all. The *fact* is that there are human beings, equivalent to the *fact* that apples fall from trees – both are seen clearly by every observer. The falling apples can be explained either by Newton’s or by Einstein’s *theory;* the existence of human beings can be explained either by Darwin’s *theory* or by direct *creation by God.* Neither is experimentally proven. However, Darwin’s theory, being within the domain of science, ought to be experimentally proven, otherwise it remains a speculative academic exercise; creation by God cannot – and need not – be experimentally proven, being part of a religious belief, and not negated by any valid scientific fact.

Dr Jacob admits that the origin of life or of a common ancestor is beyond evolution theory. I certainly agree with this statement. However, it ought to lead any serious thinker – scientist or otherwise – to ask the questions: Then how did it all start? How did life start? How did the common ancestor start? How did the Big Bang happen? There are various approaches to answer these questions. One way is to say: Science, through the theory of evolution, deals with what has happened after the common ancestor was – mysteriously – created; however, in the future, science, in some as yet unknown way, will be able to discover how it all started. Another way is to say: Science does not deal with this question; therefore, the question does not exist. These obviously are very simplistic and problematic responses to a very serious question. Yet another approach is to say: Since the origin of life is beyond the sphere of science, one can believe that it was – or at least might have been – created by a power beyond any scientific comprehension. Such an answer is based upon systems of religion and faith, which can neither be proven or negated by scientific methods. Accepting this approach actually puts one in the category of a “fundamentalist creationist”, which Dr Jacobs apparently finds to be repulsive. In any event, Dr Jacob chose to avoid this difficult problem by a meaningless statement: “To put it mildly, this is a rather odd statement for a biologist”. It might be odd to a biologist, but it certainly is not odd to any sincere and serious thinker.

Dr Jacob further argues against the concept of the survival of the fittest. Assuming that many or even most of the current scientists agree with Dr Jacob’s views on this matter, it once again proves the fact that accepted theories and concepts are changing with time. Darwin himself used this term from the fifth edition of *On the Origin of Species,* published in 1869 and on. Whatever his understanding of the term might have been, it was widely used in the strictest sense of survival of the fittest not only by natural scientists but also by sociologists, psychologists, and even politicians. Neo-Darwinism has indeed modified the concept, and perhaps even eradicated it altogether, but it again demonstrates the volatility of scientifically unproven concepts and theories.

In the section on “The ‘Jewish faith’ and the theory of evolution” Dr Jacob challenges my approach that religion believes in absolute truths whereas science is an objective method with inherent limits and with constant changes. I know of no serious philosophers of religion and science that would argue with my general statement. To a true religious believer the fundamentals of the *religion –* not the scientific quotes – are absolute truths, whereas for a serious and sincere scientist it is obvious that scientific theories and principles are objective, being valid as long as they are accepted by the current experts in the particular area of science, but they are constantly changing and often rejected with advancements in scientific and technological comprehension. Indeed, Dr Jacob describes at length the change in the very definition of “Science” itself, from what was understood and accepted beyond doubt by all scientists in the Middle Ages to what is understood by current scientists. Was science in the Middle Ages an absolute truth? Is current science an absolute truth? Can Dr Jacob be sure that what happened to the science of the Middle Ages in its most fundamental comprehension will not happen to current science? Dr Jacob is mistaken by assuming that my statement is intended to attempt to protect the truth of the Torah by casting doubt on the certainty of science. Rather, it defines the boundaries of the two sets of human thought – religion and science – which are fundamentally different in their aims and purposes, in their methods of operation, in their scope of interest and issues, and in their origin and ramifications. Hence, “Whenever science surpasses its limits, or religion exceeds its boundaries, it actually is a form of an abuse of both. This has happened to the theory of evolution in a more powerful mode than any other interaction between science and religion”.^1^

Dr Jacob goes on to discuss fundamental theological differences between his comprehension of Jewish faith and mine. Dr Jacob’s religion as described in this article is definitely not my way of understanding our religion. I certainly hope that Dr Jacob does not propose to impose his understanding of Judaism upon others. Such a paternalist approach would be absolutely intolerable. It is beyond the scope of this article to argue and reject Dr Jacob’s theological statements, which in my view are mistaken. In my article I discussed the question whether or not the accepted Orthodox perception of the Jewish faith and the theory of evolution can coexist. Dr Jacob’s way to reconcile between Jewish faith and the theory of evolution is to blindly accept the theory of evolution and completely ignore the very significant scientific difficulties with major parts of this theory on the one hand, and significantly modify Jewish religion to the degree that nothing in the theory of evolution would contradict it. This approach is serving both science and Judaism in a misleading and dishonest way.

Dr Jacob cites Maimonides as stating that if science would demonstrate evidence that directly contradicts Jewish theology we would have to change our beliefs accordingly. Indeed, in my article I quoted another, earlier, Jewish philosopher stating that the Torah can never contradict scientifically proven matters (Rabbi Halevi, 1075(?)–1140(?)).^3^ Indeed, in my article I stated that “the various details in the Biblical story of creation which appear to contradict the scientifically validated portions of the theory of evolution need not be understood literally. Most authoritative Jewish scholars agree that the technical details concerning the creation of the universe, as well as the physical-chemical processes that govern the world, are not necessarily fixed according to the literal wording in the Bible. Hence, there are different opinions and approaches concerning the manner in which God created the universe, the timing of the creation, and His degree of involvement in nature. All these issues can be interpreted in a way compatible with the *facts* of the theory of evolution”. Indeed, already the Talmudic sages and subsequent commentators of the Bible understood the verses of the beginning of Genesis not in their literal meaning. In my article I gave numerous examples to this fact. This happened hundreds of years before the theory of evolution came into existence and hence, obviously, unrelated to it. However, Maimonides, as quoted by Dr Jacob, states very clearly: “For if the Creation had been demonstrated by *proof* ... if on the other hand Aristotle had a *proof* ...” Maimonides understood very clearly that scientific *theories* are far from sufficient to disprove religious beliefs; only scientific *proof* can do so. This is exactly my argument in my article: Those parts of the theory of evolution that have been scientifically proven should be – and are – accepted by Jewish theology, even if it requires some adaptations of the Biblical and Talmudic statements: “No scientifically proven *facts* negate these statements. Moreover, all experimentally proven facts of the theory of evolution, as well as some of its assumptions and interpretations, are compatible with and accepted by Judaism”. However, major parts of the theory of evolution are unproven, speculative assumptions and therefore do not fulfill Maimonides’ criteria for theological revision.

Dr Jacob challenges my Talmudic examples on changes in nature. However, in my article I referred to the concept used by Jewish scholars, “nature has changed”, only to point out that the basic concept of intraspecies changes was well accepted in Jewish thought and law. Also, in my article I referred to extinct species described in ancient Jewish sources to point out the fact that the findings of fossils of extinct creatures were well known and accepted in ancient Jewish sources. It was beyond the scope and the purpose of my article to discuss the specific examples.

Dr Jacob concludes his article by stating the following: “After 150 years of the most intense analysis, debate, and critical testing, the theory of evolution stands as strong as ever with thousands of facts as its empirical base ... Whether we like it or not, biology simply means evolution”. In my view, such bombastic statements do not make them right or true. In fact, such a statement indeed conceals and covers up many fundamental and unresolved difficulties with the theory. Firstly, I assume that even Dr Jacob would admit that the theory of evolution itself underwent a significant evolution within the 150 years of its existence. I assume that Dr Jacob agrees with some of the observations I cited in my article: “Various fundamental scientific principles that are today accepted without dispute were rejected for many years by the scientific community because of the ‘danger’ that accepting them might cast doubts on the whole theory. Furthermore, in the early years, various ‘scientific’ theories were offered to strengthen Darwin’s theory, and even scientific forgeries were given to justify the theory of evolution”; “In the beginning of the 20th century various basic assumptions of the original Darwinian theory were found to be scientifically invalid, and the theory was in disarray. In the 1940s the evolutionists recruited scientists from different disciplines in order to revive and modernize Darwin’s theory. They proposed fundamental changes and developed the modern synthetic theory of evolution, currently known as the neo-Darwinian theory”. How then can Dr Jacob be so sure that this revised theory will withstand history without further major changes? Secondly, in my article I reviewed numerous scientific problems casting doubt on major parts of the theory of evolution, but Dr Jacob chose to ignore them completely. It is obviously legitimate for Dr Jacob to *believe* in the entire theory of evolution as such and to disregard any serious scientific criticism of major parts of it, but that he can do as a private person, not as a scientist.

Finally, Dr Jacob refrains from dealing with the argument in my article that “there is a difference between the *biological* theory of evolution, which portrays the natural evolution, and the extrapolation of this theory to the spheres of beliefs, human behavior, values, and ethics. Some scientists have expanded the biological theory of evolution into a type of a ‘religion’, explaining the universe and the psycho-ethical and political behavior of Man on the basis of beliefs and speculations which are not experimentally proven and indeed cannot be proven by scientific methods, and hence are beyond the scope of science”. Such troublesome extension of parts of the theory of evolution is based on the acceptance of the supposition that the evolution of Man from apelike creatures was accidental without purpose or intent; hence, leading to the conclusion that Man’s creation was without a goal and without a plan. “Therefore, it may lead to the conclusion that people have a right to ignore the ethical and moral foundations of humanity. This attitude is the basis for the theory of the stronger races having dominion over lower ones in accordance with the randomness of natural selection and survival of the fittest over the weakest in society. Judaism totally rejects all the extensions of the theory of evolution beyond natural sciences.” In my view it would be wrong to hide behind an argument that biological scientists have no responsibility toward other scientific fields that use – or rather abuse – unproven natural theories that are blindly supported by biological scientists.
